# Modulated release from implantable ocular silicone oil tamponade drug reservoirs

**DOI:** 10.1002/pola.28973

**Published:** 2018-02-09

**Authors:** Helen Cauldbeck, Maude Le Hellaye, Tom O. McDonald, Mark Long, Rachel L. Williams, Steve P. Rannard, Victoria R. Kearns

**Affiliations:** ^1^ Department of Eye and Vision Science University of Liverpool Liverpool L7 8TX United Kingdom; ^2^ Department of Chemistry University of Liverpool, Crown Street Liverpool L69 7ZD United Kingdom; ^3^ Unilever Research & Development Port Sunlight Laboratory, Quarry Road East Bebington Wirral CH63 3JW United Kingdom

**Keywords:** all‐trans retinoic acid, non‐polar drug reservoir, ophthalmology, PDMS, retinal detachment, silicone oil

## Abstract

Complicated cases of retinal detachment can be treated with silicone oil tamponades. There is the potential for silicone oil tamponades to have adjunctive drug releasing behaviour within the eye, however the lipophilic nature of silicone oil limits the number of drugs that are suitable, and drug release from the hydrophobic reservoir is uncontrolled. Here, a radiometric technique was developed to accurately measure drug solubility in silicone oil and measure release into culture media. All‐trans retinoic acid (atRA), a lipophilic drug known to act as an anti‐proliferative within the eye, was used throughout this work. Chain‐end modification of polydimethylsiloxane with atRA produced a polydimethylsiloxane retinoate (PDMS‐atRA), which was used as an additive to silicone oil to modify the solvent environment within the silicone oil and the distribution coefficient. Blends of PDMS‐atRA and silicone oil containing different concentrations of free atRA were produced. The presence of PDMS‐atRA in silicone oil had a positive effect on atRA solubility and the longevity of release *in vitro*. The drug release period was independent of atRA starting concentration and dependent on the PDMS‐atRA concentration in the blend. A clinically relevant release period of atRA over 7 weeks from a silicone oil blend with PDMS‐atRA was observed. © 2018 The Authors. Journal of Polymer Science Part A: Polymer Chemistry Published by Wiley Periodicals, Inc. J. Polym. Sci., Part A: Polym. Chem. **2018**, *56*, 938–946

## INTRODUCTION

Retinal detachment (RD) is a potentially blinding condition which has become one of the most common ophthalmic emergencies in the developed world.^1^ Global studies report annual incidence rates ranging from 6.3 to 18.2 per 100,000 population,^2^, ^3^, ^4^, ^5^ as well as an increase in incidence in recent years.^4^, ^6^

RD is the separation of the inner neuroretina from the supporting retinal pigment epithelium monolayer (RPE).^6^ Rhegmatogenous retinal detachment (RRD) is the most common form and occurs when there is a tear in the retina.^7^ Retinal tears can be triggered by numerous causes including blunt ocular trauma, genetic factors, myopia, inflammatory eye diseases, retinal disorders including age‐related macular degeneration, complications following cataract surgery and ageing.^1^, ^6^, ^8^, ^9^, ^10^

Proliferative vitreoretinopathy (PVR) is the leading form of blindness following retinal detachment. It occurs in approximately 5–10% of all RRD and re‐detachment after surgery occurs in 75% of these cases, making it the most common cause of failure of RRD surgery.^11^, ^12^ PVR results from a fibrotic progression of cellular proliferation, extracellular matrix deposition, membrane formation, and contraction, which can lead to a recurrence of RD.^13^ The most effective way to reduce the chance of developing PVR is to reattach the retina and allow any tears to heal. To treat complex retinal tears, vitrectomy (removal of the vitreous humour) followed by the insertion of a gas or silicone oil (SIO) tamponade into the vitreous cavity may be required. The tamponade inhibits flow of aqueous fluids into the subretinal space, excluding any inflammatory factors, and supporting retinal reattachment while the tear heals.

SIO has been used clinically since 1958 as a tamponade agent due to its transparency, low toxicity, and chemical inertness within the vitreous cavity, as well as high interfacial surface energy.^14^, ^15^ It is the tamponade agent most often used when RD/tears are numerous or complicated, or where PVR is present.^16^, ^17^, ^18^ SIO is the only long‐term tamponade agent available as it does not degrade in the eye and is not absorbed into the bloodstream. This does mean, however, that surgical removal of the SIO is required 2–8 months after the initial implantation.

Despite advances in understanding the pathology, improved imaging technology and surgical equipment, PVR remains a sight‐threatening condition. It has long been proposed that drugs could be used as adjuncts to surgical treatment with the aim of reducing the scarring associated with PVR. Agents that target various stages of the PVR cycle, in particular those which inhibit the inflammation and cellular proliferation, have been investigated.^19^, ^20^, ^21^, ^22^, ^23^ Oral administration of drugs is ineffective due to the blood–retinal barrier consequently the majority of research has concentrated on delivery directly into the vitreous cavity. The presence of SIO adds an extra level of complexity. Hydrophilic drugs, and drugs (such as triamcinolone acetonide) that could be solubilised in SIO but are simply injected, will concentrate in the aqueous layer (comprising vitreous humour that has not been removed, saline used intraoperatively and aqueous humour secreted by the ciliary epithelium) surrounding the oil, which can lead to local toxicity.^24^ It can also be difficult to load sufficient drug into the oil,^25^ or to achieve release over the 6‐ to 8‐week period thought to be required to reduce the occurrence of PVR.^26^ Despite these challenges, using SIO as a drug reservoir remains an attractive approach due to its presence in the eye until removal at the end of the healing process.

All‐trans retinoic acid (atRA) has a well‐reported anti‐proliferative and anti‐scarring effect on RPE cells,^27^, ^28^, ^29^ including those isolated from PVR membranes, *in vitro*, with the need for sustained release being highlighted.^30^ atRA has also demonstrated promising *in vivo* results in reducing the severity of experimental PVR, although too high a dose can result in retinal atrophy.^31^, ^32^, ^33^ These studies indicate that controlled, sustained intravitreal delivery of atRA *via* a SIO tamponade could act as an effective adjunctive treatment for PVR.

In previously published work,^34^ we have described the challenges associated with the development of silicone oil tamponade drug delivery reservoirs, and presented a novel polymeric additive to alter the release kinetics of acid functional drugs. This utilised silicone oil‐soluble statistical graft copolymers bearing oligo(dimethylsiloxane) and oligo(ethylene glycol) side chains which allowed hydrogen bonding with the dissolved drug molecules to reduce the rate of drug release. Here, we report a second polymeric additive approach using novel end‐functional polydimethylsiloxane‐derived additives and a new radiometric approach to accurately measure drug solubility in silicone oils as well as quantify *in vitro* drug release. To promote interaction with all‐trans retinoic acid, polydimethylsiloxane underwent chain‐end modification with atRA and was blended with unmodified silicone oil. The altered environment within the oil was hypothesised to moderate the release profile of free atRA from a silicone oil tamponade into aqueous environments. Compared to previously published work where the inclusion of a hydrophilic unit slowed the release rate of drug via hydrogen bonding, this work studies an altered hydrophobic environment and the effects of drug solubility and distribution.

## EXPERIMENTAL

### Materials

AtRA was purchased from Xian Bosheng Biological Technology Co., Ltd. and used as received. Tritiated atRA was purchased from American Radiolabeled Chemicals, Inc. in an ethanol solution which was dried before use. Ibuprofen (Ibu) was purchased from Tokyo Chemical Industry UK Ltd. and used as received. Trimethylsiloxy terminated polydimethylsiloxane oil (silicone oil; SIO_1000_; viscosity = 900–1200 cSt at 25 °C, 37,000 gmol^−1^ and SIO_5000_; viscosity = 4800–5500 cSt at 25 °C, 65,000 gmol^−1^) was donated by Fluoron GmbH and used as received. All deuterated solvents were purchased from Sigma‐Aldrich and used as received; apart from CDCl_3_, where 0.1% TMS was added. All solvents used were analytical grade and purchased from Fisher Scientific. Resazurin sodium salt was purchased from Sigma and used as received. Alexa Fluor® 488 Phalloidin (Phalloidin) and 4′,6‐diamidino‐2‐phenylindole dihydrochloride (DAPI) were purchased from Invitrogen and diluted in methanol or deionised water following the manufacturer's instructions. Adult retinal pigment epithelial (ARPE‐19) cells were bought from American Type Culture Collection, Manassas, VA, USA, catalogue number CRL 2302 and frozen stocks were stored in‐house. Dulbecco's Modified Eagle Medium/Ham's Nutrient Mixture F‐12 Formulation (DMEM/F12, catalogue number D8437), Penicillin Streptomycin 10 mg mL^−1^ streptomycin in 0.9% NaCl (Pen‐Strep), Amphotericin B solution 250 μg mL^−1^ in deionised water, Dulbecco's calcium and magnesium free phosphate buffered saline (PBS), Trypsin‐EDTA containing 5 g porcine trypsin and 2 g ethylenediaminetetraacetic acid (trypsin) and neutral buffered formalin (NBF) were purchased from Sigma‐Aldrich and used as received. Foetal bovine serum (FCS) was purchased from BioSera and used as received. All tissue culture plates and flasks were purchased from Greiner, except black 96‐well plates which were purchased from Costar. Bis(hydroxyalkyl) terminated poly(dimethylsiloxane), (*M*_n_ = 4700 g mol^−1^) was purchased from Sigma‐Aldrich and used as received.

### Measurement of atRA Saturation Concentration in Silicone Oil

UV–visible spectroscopy (UV–vis) analysis: saturated concentration of atRA in SIO_1000_ was measured based on a UV–vis characterisation method reported in the literature.^35^ In brief, samples of oil were collected, filtered to remove undissolved atRA then extracted using acetone. They were then dried and dissolved in an 8:2 solution of dimethyl sulfoxide (DMSO):H_2_O prior to UV–vis measurement. Concentration was calculated by comparing measured values to those obtained from a calibration curve generated from atRA solutions of known concentrations.

Radiometric analysis: radiometric analysis was utilised to determine solubility of drugs in silicone oil. Saturated solutions were prepared by mixing the drug (11.6 mg atRA or 32 mg Ibu) with a tritiated version of the drug (atRA: 10 µCi, specific activity 0.89 µCi mg^−1^; Ibu: 10 µCi, specific activity 0.31 µCi mg^−1^) in ethanol (EtOH, 2 mL); after evaporation of the solvent at ambient temperature, SIO (SIO_1000_ or SIO_5000_; 5 mL) was added to the residual solid and the solution was stirred for 2 weeks. Solutions were filtered using a syringe pump (4 mL h^−1^) and 0.45 µm polytetrafluoroethylene (PTFE) filters (SIO_1000_) or 1.0 µm followed by 0.45 µm polytetrafluoroethylene (PTFE) filters (SIO_5000_). Samples of the filtered oils (20 µL) were then solubilised in diethyl ether (8 mL) before scintillation cocktail (10 mL) was added. Radiation was then measured on a scintillation counter and saturation concentrations were determined.

### Synthesis of PDMS‐atRA

**Figure 1:**
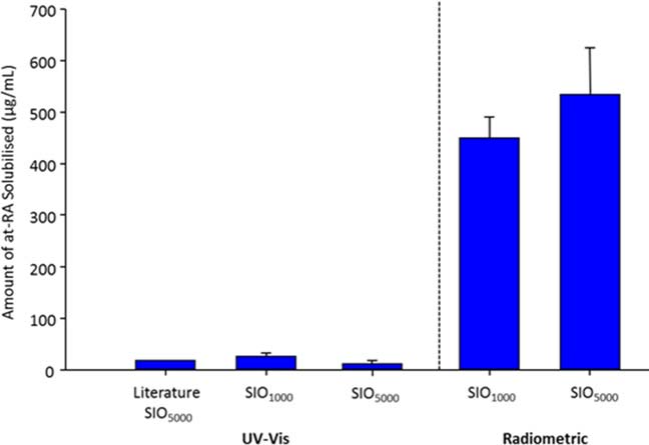
Comparison of saturation concentration of atRA measured in SIO *via* acetone extraction followed by UV–vis (SIO_1000_ *n* = 4, SIO_5000_ *n* = 3) or radioactivity measurements (SIO_1000_ *n* = 4; SIO_5000_ *n* = 4). Literature value taken from Araiz et al.^35^ Error bars +1 standard deviation. [Color figure can be viewed at wileyonlinelibrary.com.]

Bis(hydroxyalkyl) terminated poly(dimethylsiloxane), (*M*_n_ = 4700 gmol^−1^, 4 g, 1.7 mmol OH, see Electronic Supporting Information (Fig. S1) was dissolved in 10 mL of dichloromethane (DCM); oxygen was removed by gentle bubbling of nitrogen in all solutions made throughout this synthesis. A solution of atRA (0.6 g, 2 mmol) and dimethylaminopyridine (DMAP) (64 mg, 0.5 mmol) in DCM (30 mL) was added. Finally, a solution of *N*,*N*’‐dicyclohexylcarbondiimide (DCC) (0.41 g, 2 mmol) in DCM (10 mL) was slowly added. This mixture was stirred at room temperature, under argon (Ar), in the dark, for 4 days. After filtration of the residual dicyclohexyl urea, the solvent was eliminated under reduced pressure. The residual yellow oil was washed three times with cold methanol then dried *in vacuo*. The purified yellow oil was filtered through a 0.45 µm PTFE filter, then stored under Ar in dark conditions at ambient temperature. A PDMS‐atRA was analysed by ^1^H NMR and FTIR (see Fig. 1 and ESI Figs. S2 and S3). IR: 1714 cm^−1^ υ(C=O) in ester.

### PDMS‐atRA Ester Bond Stability

To determine the ester bond stability, PDMS‐atRA (70 mg) was dissolved in THF or dioxane (2.3–2.5 mL). 0.2 mL of an aqueous solution of KOH, NaOH, or DMAP, or the appropriate volume of a 1 M HCl solution was added up to 10 equivalents per ester functionality. The solution was stirred at either 40 °C or 60 °C for up to 13 days. The reaction was stopped *via* addition of DCM and the solution neutralised by distilled water washes. The organic phase was recovered, dried over Na_2_SO_4_ and filtered. The solvent was eliminated under reduced pressure, and the recovered product was dried overnight in a vacuum oven (40 °C). Residues were then analysed by ^1^H NMR spectroscopy.

### Distribution Coefficient

A blend of PDMS‐atRA in SIO_1000_ at 10% volume content of PDMS‐atRA was prepared and mixed in a sealed container in the dark for approximately 4 days. Saturated solutions of atRA in either SIO_1000_ or the blend of SIO_1000_ with PDMS‐atRA were prepared using a mixture of drug and tritiated drug, and analysed using the same radiometric method as described above. Saturated solutions were prepared as above. The shake‐flask method was used to determine the distribution coefficient of atRA in SIO_1000_/PDMS‐atRA blend and media the shake‐flask method was used. SiO_1000_ (3 mL) or PDMS‐atRA blend (10 v/v %) was placed over media (3 mL) in a 20 mL vial and agitated. The vials were left at 37 °C for 2 weeks to reach equilibrium and radiometric analyses performed to determine atRA concentration in each phase.

### atRA Solubility in and Release Profile from PDMS‐atRA Blends

Blends of PDMS‐atRA and SIO_1000_ at 1%, 5%, and 10% volume content of PDMS‐atRA were prepared and mixed in a sealed container in the dark for approximately 4 days. Saturated solutions of atRA in either SIO_1000_ or blends of SIO_1000_ with PDMS‐atRA were prepared using a mixture of drug and tritiated drug, and analysed using the radiometric method described previously. Amounts of atRA added to the samples were altered depending on targeted final concentrations. Solutions of varying concentrations of atRA (20–813 µg mL^−1^) and tritiated atRA (specific activity >890 µCi µg^−1^) were mixed in EtOH (2 mL) and the same protocol was followed. To determine release concentrations of drug, 1 mL of SiO_1000_ or PDMS‐atRA blend (1, 5, or 10 v/v %) with a determined concentration of drug was placed in a 24‐well plate over 0.5 mL culture media, see ESI Figure S4. Samples of media (0.5 mL) were taken and replaced at determined time intervals; daily for the first critical week then every 2–3 days for up to 50 days, using a 1 mL syringe and 25 gauge needle, for up to 71 days. Withdrawal and replacement of the media were done very carefully to avoid any emulsification of the oil. Media samples (250 µL) were mixed with scintillation cocktail (10 mL) and analysed by liquid scintillation counting.

### Cytotoxicity Assays of Drug Compounds and PDMS‐atRA

See ESI for details of cell culture and extended information of assays/staining performed. 18,000 ARPE‐19 cells/well were seeded in a 48‐well tissue culture plate and left for 1 or 7 days to adhere to the plate (to model pre‐confluent and confluent cells respectively). After the predetermined time period, the media were aspirated from all wells and replaced with 0.4 mL fresh media along with 0.2 mL SIO_1000_ containing PDMS‐atRA blend at 10% (v/v) or controls. Controls included: media, SIO_1000_ (0.2 mL) and a positive control (20 v/v % DMSO in media). Cells were incubated for 1–7 days before a resazurin assay (to assess metabolic activity) and qualitative assessment of cell morphology following phalloidin and DAPI staining were performed.

### Rheology

All rheological measurements were carried out using a TA Instruments Rheolyst AR 1000 N controlled‐stress rheometer (TA Instruments, Elstree, United Kingdom) in steady shear over a shear stress range of 0.5–25 Pa. A 50 mm diameter, 4° steel cone geometry was used. The cone tip is truncated by 106 µm to allow the virtual tip of the cone to coincide with the surface of opposing plate without added friction. Temperature control of the rheometer is achieved *via* a plate that utilises the Peltier effect to control the temperature of the sample within ±0.1 °C. Shear viscosity was calculated from the gradient of the plot of shear stress against shear rate. Three replicate samples for each blend were tested.

### Statistical Analyses

Statistical analyses were carried out on SPSS Statistics V22 software; one‐way test of homogeneity of variances and analysis of variance (ANOVA) as well as Dunnett's T3 post‐hoc evaluation were conducted, *p* ≤ 0.05 was regarded to be statistically significant.

## RESULTS AND DISCUSSION

### Measurements of atRA Saturation Concentration in Silicone Oil

The saturation concentrations of atRA in SIO_1000_ and SIO_5000_ when using an extraction, combined with UV–vis spectroscopy (UV–vis) characterisation method were determined as being 28.5 µg mL^−1^ and 14.7 µg mL^−1^, respectively (ESI Figs. S5 and S6). The data represent a 42.5% increase (SIO_1000_) and 26% (SIO_5000_) decrease over the reported value (20 µg mL^−1^), although in the previous report, only SIO_5000_ was used to obtain this value.^35^ Extraction separates the substance of interest from a mixture, this involves the use of two immiscible solvents (in this case SIO_1000_ or SIO_5000_ and ethanol); the desired compound (here atRA) in suspension in one solvent is then extracted into the other solvent. In this study, the solvent was then removed and the substance of interest dissolved in a second solvent; UV–vis measurements were then taken. Concentrations were calculated from a previously established calibration curve (ESI Fig. S5). These multiple steps can result in inaccurate measurements due to drug loss and degradation. It is important to note that the method reported previously required considerable extrapolation from the achievable calibration curve, adding potential errors to the values claimed within the manuscript. In addition, limitations of direct drug release measurement were observed in this study when studying drug release from SIO into cell culture media, as a strong overlap of UV–vis absorption peaks of the drug compound and media components, such as foetal calf serum, was seen (ESI Fig. S7). Attempts to accurately quantify the concentration of atRA in silicone oil directly by ^1^H nuclear magnetic resonance (NMR) spectroscopy of the oil were impeded by the low solubility of atRA in this liquid. To overcome the errors associated with these limitations, the use of radiolabelled ^3^H‐atRA was investigated. Mixtures of atRA with relatively low ratios of radiolabelled ^3^H‐atRA were used, allowing accurate measurement of silicone oil samples containing very low drug concentrations, without subsequent manipulation and analytical errors. In addition, ^3^H‐labelled atRA is essentially unchanged chemically from its unlabelled counterpart and the use of techniques to amplify detection that require conjugation of fluorophores are avoided.^36^, ^37^ The saturation concentration measured after filtration was over 20 times higher than the corresponding values determined by UV–vis spectroscopy for SIO_1000_, 450.6 µg mL^−1^ (1.5 × 10^−3^ M, *n* = 4) and 529.1 µg mL^−1^ (1.8x10^−3^ M, *n* = 4) for SIO_5000_ (Fig. 1).

The accuracy of the radiometric technique was validated by the use of an alternative drug, Ibuprofen, which is known to have a much higher saturation concentration in silicone oil, that is approximately 2 mg mL^−1^, therefore allowing comparative measurements by both radioactivity and ^1^H NMR directly on the oil solutions. The saturation concentration values measured for Ibu in SIO_1000_ using either radiometric analysis or NMR were in good agreement, with measured values being within 10% (ESI Fig. S8).

The use of radioisotopes provided a much more accurate method to measure very low drug concentrations in SIO compared to previously reported UV–vis approaches. Furthermore, the difficulty of extracting drug from highly viscous oils such as SIO_5000_ is overcome.

### *In Vitro* Release of atRA from SIO1000

A clinically relevant treatment period for PVR therapy lies in the range of 6 weeks,^26^ however, it has previously been reported that 10 µg mL^−1^ atRA administered *in vivo via* an SIO tamponade has a 98% clearance within a 7‐day time period.^35^

Here, the release of atRA from SIO_1000_ into culture media was monitored using tritiated atRA and a series of experiments was performed with different atRA starting concentrations, from 32 µg mL^−1^ to >400 µg mL^−1^, the saturation concentration of atRA in SIO_1000_. As evidenced by the representation of the cumulative percentage of atRA released over time (see Table 1), the cumulative release profile is very similar for all the concentrations studied, with 80% of the total amount of drug released from SIO_1000_ after 14 to 16 days.

**Table 1:** Number of Days Needed to Reach a Certain Percentage (Cumulative) of atRA Released from SIO_1000_ Containing Different Initial Concentrations of atRA

	Days Taken to Reach Percentage of Release		
Cumulative Percentage	32 µg mL^−1^	49.3 µg mL^−1^	412.5 µg mL^−1^
10	<1	<1	<1
20	<1	<1	<1
30	1.2	1.8	1.6
40	1.8	2.3	2.4
50	2.6	3.1	3.3
60	4.4	5.1	5.4
70	8.3	8.7	8.2
80	14.9	16.2	14.0
90	25.0	–	35.0
100	–	–	–

### Synthesis and Characterisation of PDMS‐atRA

It was hypothesised that the use of an additive to SIO would generate a sustained drug release over clinically relevant periods. End‐functionalised PDMS was chosen as an additive to the model tamponade due to its chemical similarity to the surrounding SIO. This similarity led to complete miscibility with SIO_1000_; additionally, in the event of unexpected atRA cleavage and release from the chain‐ends, the remaining polymer backbone would be nearly identical to the surrounding SIO.

PDMS‐atRA (1) was synthesised using the Steglich esterification method.

The commercially available bis(hydroxyalkyl) terminated poly(dimethylsiloxane) was reacted with atRA, at ambient temperature in dichloromethane (DCM). These conditions were tested beforehand to establish atRA stability to the reaction conditions and no degradation was observed after 4 days (see ESI Fig. S9). The working concentrations of reactants were based on the maximum solubility of atRA in DCM determined previously (i.e., 12 mg mL^−1^).

**Figure 2:**
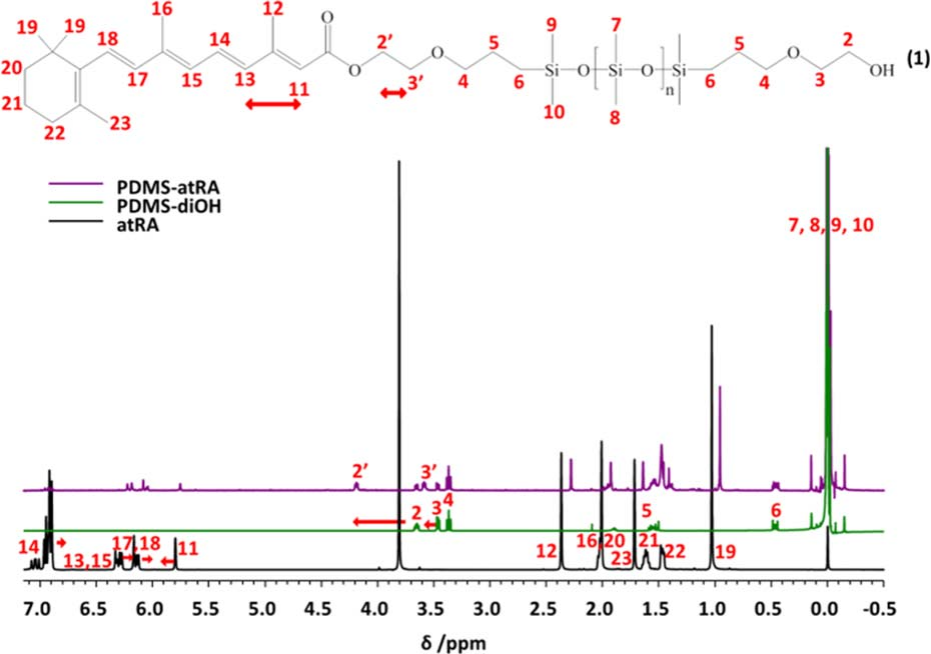
^1^H NMR spectra (CDCl_3_, 400 MHz) of starting PDMS‐diOH (green) and atRA (black) along with PDMS‐atRA (purple) after purification. [Color figure can be viewed at wileyonlinelibrary.com.]

The formation of PDMS‐atRA was monitored by ^1^H NMR spectroscopy (see Fig. 2) with the formation of the ester bond characterised by the appearance of a peak at 4.31 ppm (labelled 2′), as well as a chemical shift of all the signals of the protons close to both the acid function of the drug (e.g., 11, 12, 13, and 14) and the hydroxyl function of PDMS‐diOH (e.g., 3). This classic esterification reaction usually requires no longer than 24 h to achieve completion, but this depends on the nature of the acid and alcohol involved in the reaction.

For atRA the reaction was much slower, reaching only 40% conversion after 2 days, and usually less than 70% after 4 days (*n* = 4, ESI Table S1). Analysis by ^1^H NMR revealed that 58% of PDMS‐diOH terminal groups were functionalised with atRA (see ESI Fig. S1). Due to the equal reactivity of the hydroxyl groups it is expected that both mono‐ and di‐esters as well as some unreacted precursor is present in the final product. The abundance of each of these species cannot be determined *via* ^1^H NMR, therefore a statistical distribution between the three species has been assumed.

The conjugation throughout atRA stabilises the acid function by making the carbonyl function less available to react. Leaving the reaction for more than 4 days did not lead to higher conversions, therefore, all reactions were stopped at 4 days to limit degradation of atRA. The final products were purified by repeated washes in MeOH, allowing for the removal of unreacted atRA. ^1^H NMR shows this purification process involves the loss of some of the shorter PDMS chains within the molecular weight distribution and addition of atRA, therefore, leading to a higher *M*_n_ value of 7100 g mol^−1^ within the final products than the initial starting material of 4700 g mol^−1^. PDMS‐atRA was fully characterised by ^1^H and ^13^C NMR spectroscopy, as well as FT‐IR spectroscopy which confirmed the formation of an ester bond, with the appearance of a characteristic ester signal (see Fig. 2, ESI Figs. S2, S3, and S10).

### Stability of PDMS‐atRA

The stability of PDMS‐atRA and the integrity of the ester bond were investigated under different conditions (Table 2). While the different conditions tested (mainly strong basic and acidic conditions) are not particularly relevant *in vivo*, they highlighted the robustness of the ester linkage between the PDMS and the drug. Indeed, none of the conditions tested (up to 10 equivalents of HCl per ester function, at 40–60 °C) allowed for the cleavage of the ester bond, as shown by ^1^H NMR analyses (ESI Figs. S11–S13). While the PDMS chains suffered some decomposition in the presence of 2–5 equivalents of KOH, evidenced by both ^1^H NMR and GPC analyses (ESI Figs. S14 and S15), it remained intact under all the other conditions. In most of the experiments, atRA suffered some degradation while the ester linkage remained. Following these results it is likely in any physiological condition, even during infection when pH is increased, that the ester bond would remain and the drug would not be cleaved from the additive therefore no drug is released unless free drug is present.

**Table 2:** Conditions Used to Attempt the Cleavage of the PDMS‐atRA *In Vitro* and Their Effects on PDMS and atRA. x = No Cleavage, Degrades PDMS or Degrades atRA. ✓ = PDMS/atRA Stable

Solvent	Time (h)	Condition	Ester Cleaved	PDMS Stable	atRA Stable
THF (40 °C)	22	2 eq. KOH	x	x	x
	22	5 eq. KOH	x	x	x
	312 (13 days)	2 eq. DMAP	x	✓	x
	168 (7 days)	5 eq. DMAP	x	✓	x
	312 (13 days)	2 eq. DMAP with H_2_O	x	✓	x
	168 (7 days)	5 eq. DMAP with H_2_O	x	✓	x
	24	5 eq. HCl	x	✓	✓*
	24	10 eq. HCl	x	✓	✓*
Dioxane (40 °C)	24	5 eq. HCl	x	✓	✓*
	9	10 eq. HCl	x	✓	✓*
Dioxane (60 °C)	9	5 eq. HCl	x	✓	x
	6	10 eq. HCl	x	✓	x
Stabilised dioxane (60 °C)	9	5 eq. HCl	x	✓	x

✓* No clear degradation but decrease in signal intensity in ^1^H NMR.

### Effects of PDMS‐atRA Blend on Distribution Coefficient

The distribution coefficient is the ratio of solubility of a compound in a mixture of two immiscible phases at equilibrium. In this instance the two phases consisted of media and SIO_1000_ or PDMS‐atRA blend in SIO_1000_ 10 v/v %. The distribution coefficient determined that PDMS‐atRA 10 v/v % was more lipophilic than SIO_1000_ that is more atRA remained in the blend than SIO_1000_ (see Table 3)_._

**Table 3:** Determination of Distribution Coefficient of atRA in Media and SIO_1000_ or PDMS‐atRA 10 v/v %

	SIO_1000_		PDMS‐atRA 10 v/v %	
Initial atRA (µg)	129.92		383.04	
	Media	Oil	Media	Oil
atRA (µg)	75.65	47.40	215.02	167.26
Distribution coefficient	0.627		0.778	

### Cytotoxicity of PDMS‐atRA Blends with ARPE‐19 Cells

**Figure 3:**
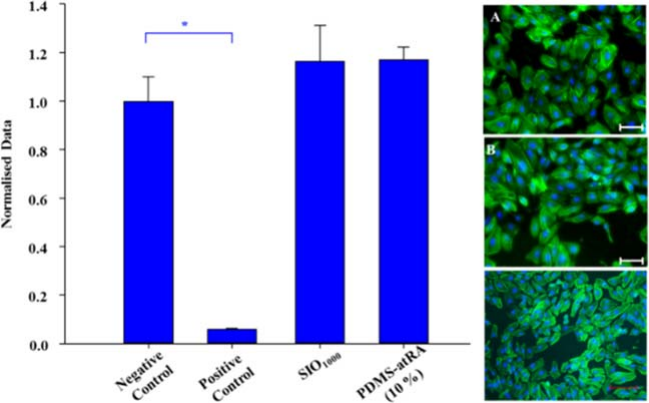
LEFT: Resazurin assay with appropriate controls to determine the cytotoxicity of SIO_1000_ and blends of PDMS‐atRA with SIO_1000_ at 10% (v/v). Pre‐confluent ARPE‐19 cells (grown for 1 day) were exposed to the oils for 1 day. Data were normalised to the mean value for the negative control. Bars represent mean, error bars represent ± 1 standard deviation; *n* = 3. *Significance by ANOVA and Dunnett's T3 post‐hoc evaluation (*p* < 0.05). RIGHT: ARPE‐19 cells stained with phalloidin (green, F‐actin cytoskeleton) and DAPI (blue, nuclei). Pre‐confluent cells (grown for 1 day) exposed to A: Negative control, B: cells exposed to SIO_1000_ and C: blends of PDMS‐atRA with SIO_1000_ at 10% (v/v). Cells were exposed for 1 day. Scale bars represent 50 µm. [Color figure can be viewed at wileyonlinelibrary.com.]

There was a significant difference in the metabolic activity and morphology of ARPE‐19 cells between the negative control and the positive control. However, no significant difference was observed between the negative control and cells exposed to SIO_1000_ and PDMS‐atRA blend at 10 v/v % in SIO_1000_, indicating that the oil and blends have no cytotoxic effect on the ARPE‐19 cells (Fig. 3). Cells exhibited healthy cytoskeletons, also suggesting the blends had no cytotoxic effects (Fig. 3), and no qualitative differences in cell density were observed, suggesting that cell proliferation and spreading behaviours were unaffected.

### Effects of PDMS‐atRA Blends and atRA Solubility and Release Profiles *In Vitro*

The likely positive effects of PDMS‐atRA on solubility and diffusion behaviour of drug in SIO_1000_ were studied. The hypothesised affinity between drug molecules and PDMS‐atRA gives the potential for these materials to act as additives which would modify the release and solubilisation behaviour of drug from SIO. The same radiometric protocol used to determine accurate atRA solubility in SIO was employed to determine free atRA concentration in blends of PDMS‐atRA in SIO_1000_ at 1%, 5%, and 10% (v/v). As the amount of PDMS‐atRA present in the blend increased so did the measured saturation concentration of atRA (see Table 4). An increase from 413 µg mL^−1^ to 813 µg mL^−1^ was observed when a blend of 10% PDMS‐atRA in SIO_1000_ was used compared to SIO_1000_ alone. This indicated the blends have a strong effect on the solubility of the free drug. The saturation concentration of free drug in the oil almost increases by a factor of 2. As hypothesised the additive changes the solubility parameter of SIO_1000_ this was also demonstrated by the increased lipophilicity of the blend PDMS‐atRA 10 v/v % in SIO_1000_ as shown by the increase in distribution coefficient within the oil phase.

**Table 4:** Saturation Amounts of atRA (µg mL^−1^) in SIO and SIO Blended with PDMS‐atRA at 1%, 5%, and 10% (v/v) After 2 Weeks at Ambient Temperature, in Air

	SIO	1% PDMS‐atRA	5% PDMS‐atRA	10% PDMS‐atRA
atRA (µg mL^−1^ oil)	413	651	700	813

**Figure 4:**
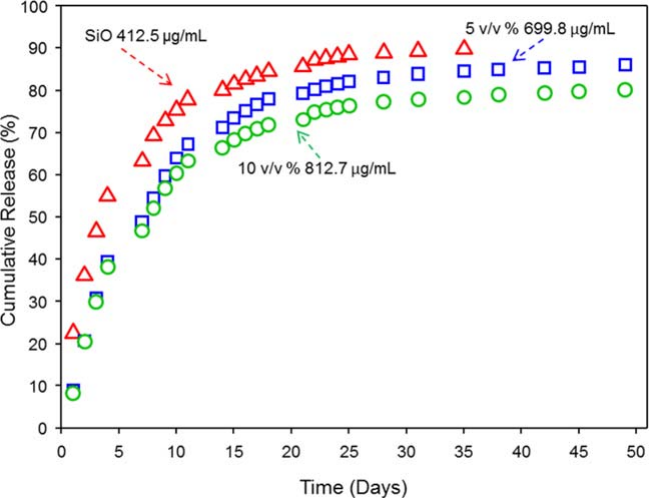
Release amounts of atRA from saturated SIO1000 (open red triangles), 5 (open blue squares), and 10 (open green circles) % (v/v) blends of PDMS‐atRA in SIO_1000_ into culture media. [Color figure can be viewed at wileyonlinelibrary.com.]

The release of atRA from SIO_1000_ and SIO_1000_ blends with PDMS‐atRA into culture media was monitored using radiometric analysis. A rapid release of atRA is observed within the first few days (Fig. 4), after which the release gradually slows and then becomes steady over the remaining 20 days. The burst release is reduced with addition of the additive PDMS‐atRA.

When looking at the amount of atRA released from SIO and the blends as a cumulative percentage (Table 5), it is very interesting to note the difference in time taken for 80% (the maximum cumulative percentage release achieved for all three oils over the duration of the study) of the solubilised atRA to be released: 2 weeks for SIO_1000_ compared to nearly 7 weeks for the 10 v/v % blend. This suggests that the presence of the blends may extend the sustained release into the 6‐week period that is required clinically. This differs from the literature reports of 98% atRA release from a saturated SIO_1000_ solution over 7 days (using a rabbit model)^35^ which may be due to the errors identified here with the extraction/UV–vis assay protocol employed in those studies. Other important differences between that report and the data reported here are the increased clearance in a rabbit eye due to elimination *via* aqueous humour outflow into the anterior chamber of the eye and *via* various retinal routes, and increased oil:aqueous in a vitrectomised eye compared to the *in vitro* setup used here. Other important differences between that report and the data reported here are the increased clearance in a rabbit eye due to elimination *via* aqueous humour outflow into the anterior chamber of the eye and *via* various retinal routes, and increased oil:aqueous in a vitrectomised eye compared to the *in vitro* setup used here. While very promising release over previously unreported long time periods were observed, the concentrations of atRA released at early time points (7 days for SIO_1000_ and 9 days for both PDMS‐atRA blends) were above the cytotoxic range for ARPE‐19 cells (0.03–0.3 µg mL^−1^) when starting from saturated solutions. To study the effect of the different blends on the drug release profile for lower starting concentrations of atRA a series of release experiments with similar amounts of atRA (around 50 µg mL^−1^) in SIO and blends with PDMS‐atRA were conducted. This was to confirm the extension of the release period due to the use of blends, and to attempt to avoid any potential cytotoxicity upon release over the first few days. The time needed to release 80% of the atRA solubilised in SIO_1000_ was on average 16 days (∼2 weeks) whereas for the 10% blend, this increased to nearly 51 days (> three times longer, ∼7 weeks) (Table 6). This confirms that the presence of PDMS‐atRA helps extend the release profile into the 6‐week period required clinically.

**Table 5:** Number of Days Taken to Reach a Certain Cumulative Percentage of atRA Released from Saturated Solutions of SIO_1000_ and 5 and 10% (v/v) PDMS‐atRA Blends

	Days Taken to Reach Percentage of Release		
Cumulative Percentage	SIO_1000_ (412.5 µg mL^−1^)	5% Blend (700 µg mL^−1^)	10% Blend (813 µg mL^−1^)
10	<1	1.1	1.2
20	<1	1.8	1.8
30	1.6	2.9	3.1
40	2.4	4.1	4.2
50	3.3	6.8	7.2
60	5.4	9.2	9.9
70	8.2	12.8	16.4
80	14.0	21.9	48.2
90	35.0	–	–
100	–	–	–

It is interesting to notice that the initial release profiles obtained from blends is very similar regardless of the starting concentration. These observations are in agreement with the release profiles from SIO_1000_ at different starting concentrations that is 412.5 µg mL^−1^ of atRA, or a concentration one eighth of that, that is 49.3 µg mL^−1^; it is taking 14–16 days for 80% of the atRA to be released. Similarly, whether we start from a saturated solution of the 10% (v/v) blend of PDMS‐atRA in SIO_1000_, that is 813 µg mL^−1^ of atRA, or a concentration almost one eighteenth of that, that is 46.2 µg mL^−1^, it takes from 48 to 51 days for 80% of the atRA to be released. This clearly shows that the release profiles of atRA from blends of SIO_1000_ and PDMS‐atRA, as well as from SIO_1000_ alone are independent of the starting concentration of atRA present in the oil reservoir.

**Table 6:** Number of Days Needed to Reach a Certain Percentage (Cumulative) of atRA Released from SIO_1000_ and 5 and 10% Blends Containing Approximately the Same Initial Amount of atRA, that is 50 µg mL^−1^

	Days Taken to Reach Percentage of Release	
Cumulative Percentage	SIO_1000_ (49.2 µg mL^−1^)	10% Blend (46.2 µg mL^−1^)
10	<1	<1
20	<1	<1
30	1.7	1.6
40	2.2	2.4
50	3.1	4.6
60	5.1	7.9
70	8.7	15.3
80	16.2	50.8
90	59.0	–
100	–	–

Due to the release rate being independent from starting concentration, it would potentially be possible to reduce the burst release profile to a concentration below the toxic threshold and yet still maintain a sustained release. *In vivo* studies have demonstrated that doses of 5 and 10 µg atRA released from silicone oil over 7 days^35^ and doses of 420–1070 µg delivered over 8 weeks (peak dose ∼1.4 µg mL^−1^) from an intravitreal polylactic acid–coglycolic acid implant (PLGA)^38^ are effective at reducing PVR in rabbits. PVR was not avoided entirely in either case, possibly due to the short duration of drug release^35^ or the peak drug release happening after PVR formation.^38^ Neither study reports retinal toxicity, even with the highest dose, which is greater than the largest dose available from saturated 10% PDMS‐atRA blends. The PDMS‐atRA blends were able to deliver *in vitro* doses in a similar range to those reported for silicone oil (although, due to the measurement uncertainties with UV–vis discussed in 3.1, it is impossible to make a direct comparison with that work, or the work on PLGA) but over much longer, and clinically relevant time periods, and with release profiles (peak doses over the earlier time points, when PVR is initiated) that are more clinically relevant than from the PLGA implant.

### Rheology

Forces at the interface of a SIO tamponade in the eye with the surrounding aqueous phase (which is comprised of aqueous humour, vitreous humour not removed during vitrectomy, and residual intraoperative saline) can cause the disruption of the oil with breakup into droplets. These emulsions are stabilised by the presence of surfactants and cellular debris in the aqueous. This can lead to two problems: the tamponade no longer being effective and, if the droplets are small enough, postoperative complications, such as optic neuropathy and major corneal complications, may occur. It was important to study the shear viscosity of the blends and compare with SIO_1000_ as the higher the shear viscosity, the greater the shearing force required to disperse a tamponade agent into small droplets.

The shear viscosity of SIO_1000_ and the blends with end‐modified PDMS, were measured at 20 °C and 37 °C (see ESI Fig. S16 and Table S2). The measured viscosities show that there was no effect of saturating SIO_1000_ with atRA, but that adding the PDMS‐atRA did decrease the viscosity (from 0.79 mPas for SIO_1000_ to 0.56 mPa s for SIO_1000_ with 10% PDMS‐atRA). The decrease in viscosity of the blends did not follow a trend based on their composition (i.e., the amount of PDMS‐atRA), therefore, the increase in atRA saturation concentration observed with the blends is not solely a function of decreased viscosity.

## CONCLUSIONS

In this study, PDMS‐atRA has been synthesised and used as an additive for potential silicone oil tamponade applications. The ester bond within this additive has been shown to be very stable under a range of harsh chemical conditions. The presence of PDMS‐atRA in SIO had a positive effect on atRA solubility, as indicated by the diffusion coefficient, as well as a positive effect on the longevity of atRA release into aqueous media; the release period being independent of atRA starting concentration and dependent on the PDMS‐atRA concentration in the blend. Excitingly, a release period of atRA from a SIO blend with PDMS‐atRA over 7 weeks was observed and the tailoring of starting atRA concentrations may lead to control of the burst release below the toxic range for relevant cell types; the sustained release was above the reported therapeutic level for an anti‐proliferative effect to be observed *in vivo*.

## Supporting information

Supporting Information
