# Supplementary material for: Modulated release from implantable ocular silicone oil tamponade drug reservoirs
Source: J Polym Sci A Polym Chem. 2018 Feb 9;56(8):938–46. doi: 10.1002/pola.28973 (PMC5873246; doi:10.1002/pola.28973)
Supplement: Supplementary file 1 — Supporting Information [file POLA-56-938-s001.pdf]

Modulated release from implantable ocular silicone oil tamponade drug reservoirs

Helen Cauldbeck,[a,b] Maude Le Hellaye,[a,b] Tom O. McDonald,[b] Mark Long,[c] Rachel L. Williams,[a] Steve P. Rannard[b]* and Victoria R. Kearns.[a*]

[a]Department of Chemistry, University of Liverpool, Crown Street, Liverpool, L69 7ZD, UK

[b]Department of Eye and Vision Science, University of Liverpool, William Henry Duncan Building, 6 West Derby Street, Liverpool, L7 8TX, UK

[c]Unilever Research & Development, Port Sunlight Laboratory, Quarry Road East, Bebington, Wirral CH63 3JW, UK

Supporting Information

Experimental Details

Characterisation

Nuclear Magnetic Resonance (NMR) spectra were recorded using a Bruker DPX-400 spectrometer operating at 400 MHz for ^1H NMR and 100 MHz for ^{13}C NMR. UV-Vis spectra were collected using a Thermo Fisher NanoDrop 2000c spectrophotometer with a quartz cuvette. Data was analyzed using the NanoDrop2000 software. All radiation measurements were carried out using a liquid scintillation counter (Packard Tri-carb 3100TR; Isotech). Triple detection gel permeation chromatography (GPC) was performed to measure molecular weights and molecular weight distributions using a Malvern Viscotek instrument. The instrument was equipped with a GPCmax VE2001 autosampler, two Viscotek T6000 columns (and a guard column), a refractive index (RI) detector VE3580 and a 270 Dual Detector (light scattering and viscometer) with a mobile phase of tetrahydrofuran (THF) containing 2 v/v % of trimethylamine at 35 °C with a flow rate of 1 mL min^{-1}.
A Zeiss Axiovert 200 microscope system was used to collect cell images. Fourier Transform Infrared (FT-IR) spectroscopy was carried out using a Bruker Tensor 27 plate reading FTIR. For each sample, 20 scans in the region from 650 to 4000 cm^{-1} were accumulated with a resolution of 4 cm^{-1}.

Measurement of atRA in silicone oil

UV-Visible spectroscopy

A protocol reported in the literature was used;[1] a saturated solution of atRA in SIO was prepared and stirred gently in the dark at ambient temperature. Samples were taken at different time-points over a 2 week period and filtered using a syringe pump (4 mL/h) through 0.45 µm PTFE filters. The drug was extracted from the SIO three times with acetone (1:2 v/v). The acetone layers were combined and the solvent was evaporated with the remaining solid dried at ambient temperature. The samples were solubilised in 2 mL of a mixture of DMSO/H_2O (8/2) and subsequently analysed by UV-Vis spectroscopy. A standard curve previously determined for atRA (see Electronic Supporting Information; ESI; Figure S1) was used to determine the drug concentration.

Cytotoxicity assays of drug compounds and atRA-PDMS-atRA

ARPE-19 cells between passage 8 and 24 were cultured at 37 °C in a dark, humid 5 % CO_2 incubator in media containing 1 % Pen-Strep, 1 % Amphotericin B and supplemented with 10 % FCS. For these studies, cells were used between passages 22 and 25. Multiple assays were carried out to study the effects of different drug concentrations on cell viability, metabolic activity and cell morphology. 18,000 cells/well were seeded in a 48 well tissue culture plate and left for 1 or 7 days to adhere to the plate. The 7 day samples were fed once within the week by replacing 450 µL old medium with 500 µL fresh culture medium. After the predetermined time period, the media was aspirated from all wells and replaced with 0.6 mL fresh media containing polymer blends or controls. Controls included: media, SIO_{1000} (0.2 mL) and a positive control (20 v/v % DMSO in media). Cells were incubated for 1 to 7 days before the following assays could be performed.
Resazurin stock solution was prepared by dissolving resazurin sodium salt in PBS at 0.1 mg/mL, filtered and stored at 4 °C in the dark. Medium was removed and replaced with resazurin solutions (10 % v/v stock solution in medium). Plates were incubated in the dark at 37 °C for 4 hours. The resazurin solution was removed and put in black 96-well plastic plates; resorufin fluorescence was read using a Biotek FLx800 spectrofluorometer (λExcitation = 530 nm; λEmission = 590 nm). All values were normalized to negative control wells on each plate. Finally statistical analyses were performed.
Immediately following removal of resazurin solution, cells were washed with PBS (500 µL) then fixed for 10 minutes in 10 % neutral buffered formalin (NBF; 10 % formalin, approximately 4 % formaldehyde). NBF was discarded and the cells washed again with PBS. A phalloidin solution was used to stain the F-actin of the cytoskeleton of the cell. A phalloidin solution was produced at 1 mg powder in 1.5 mL MeOH according to manufacturer's instruction. The solution was then diluted 1 in 100 (in fresh PBS) and 75 µL was placed in each well followed by 30 minutes of incubation at 4 °C. Phalloidin solution was removed and the cells washed with PBS.
DAPI was used to stain the nuclei of the cell. A stock solution of DAPI was made at 1:1,000 with PBS, then further diluted to a working solution of 1:10 PBS, 75 µL was placed in each well and incubated for 10 minutes at 4 °C. Cells were washed with PBS and placed under 500 µL PBS. Cells were then imaged using fluorescence microscopy.

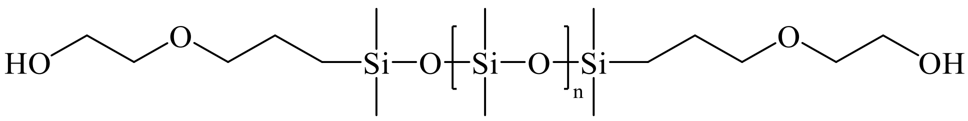

Figure S1 Poly(dimethylsiloxane), bis(hydroxyalkyl) terminated (4,700 gmol^{-1})

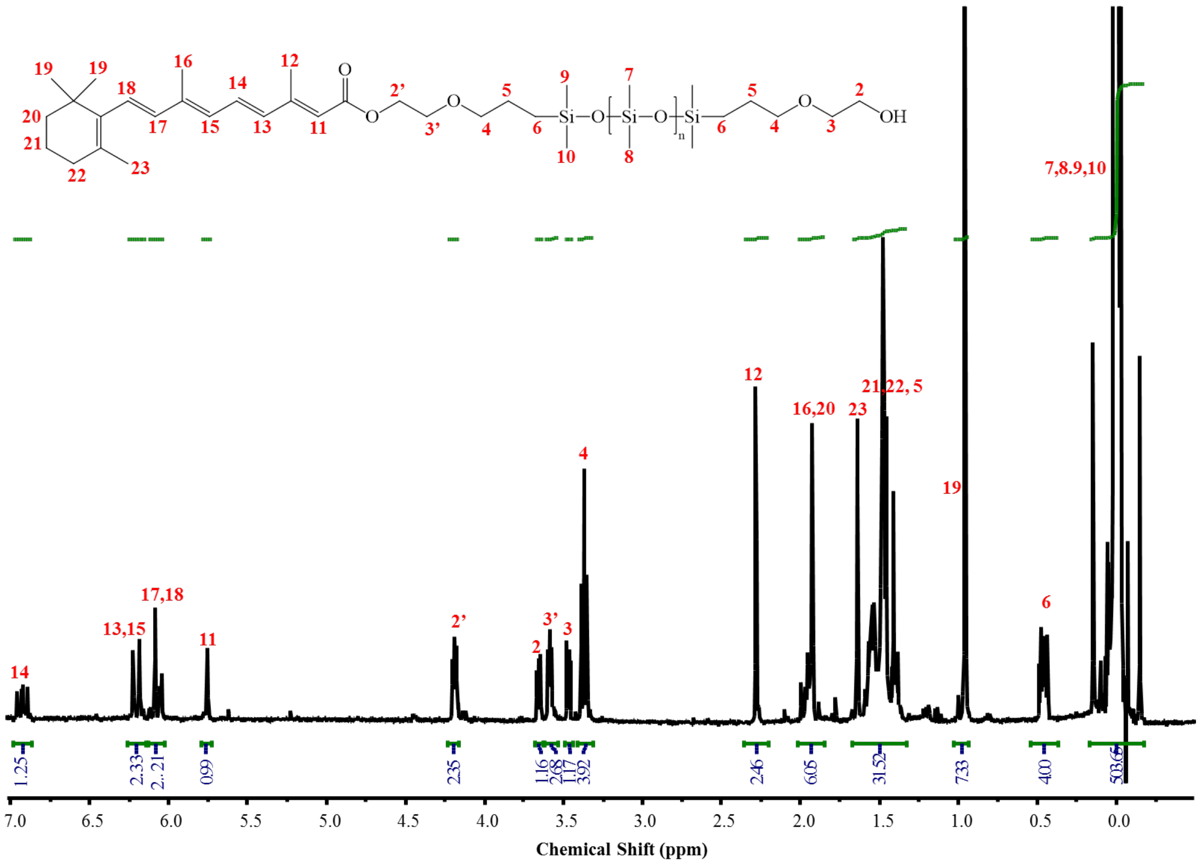

Figure S2 ^{1}H NMR ($CDCl_3$, 400 MHz) PDMS-atRA. 58 % conversion calculated. Peak at 0.47 ppm (6) normalised to 4 corresponding to Si-C<u>H</u>$_2$ and compared to peak at 4.20 ppm (2') corresponding to O-CH_2 next to ester.

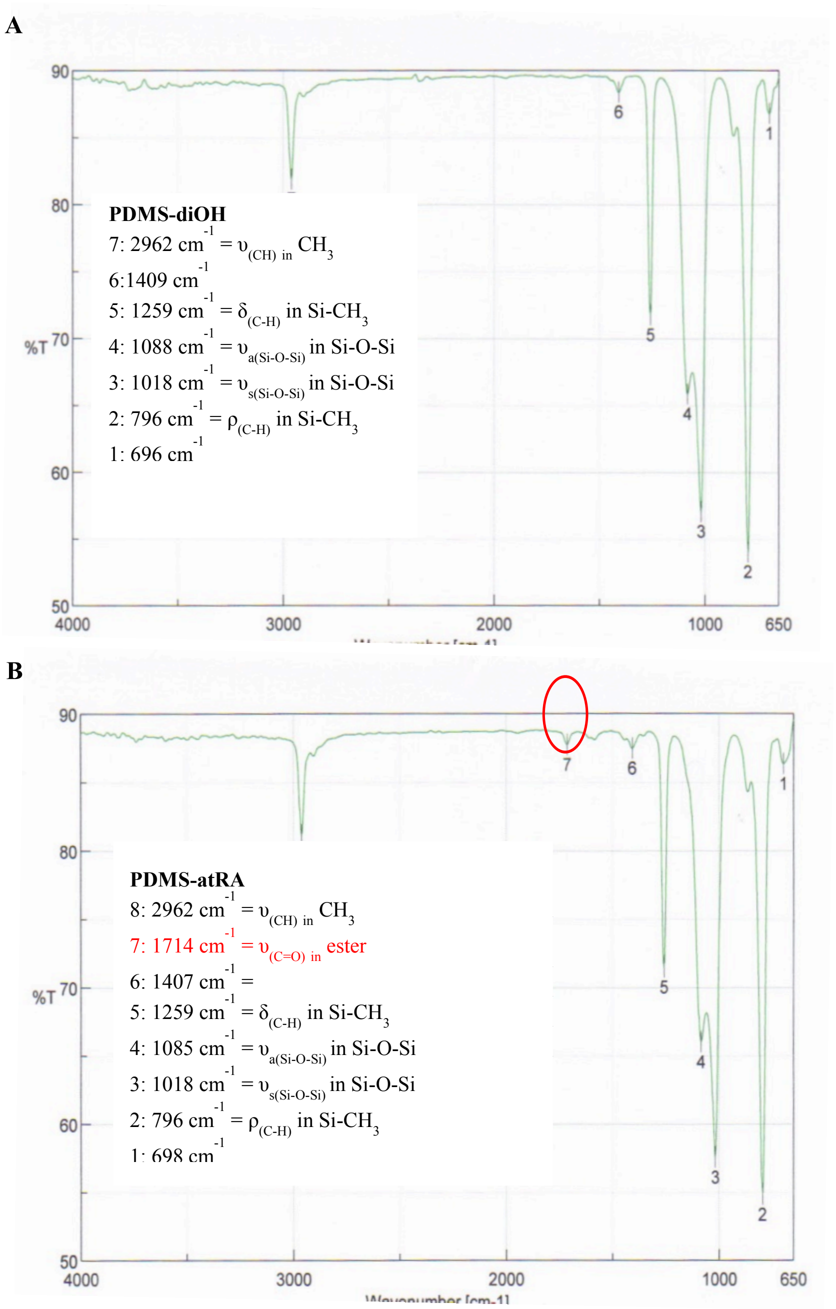

Figure S3 FTIR analysis of A: PDMS-diOH and B: atRA-PDMS-atRA after purification.

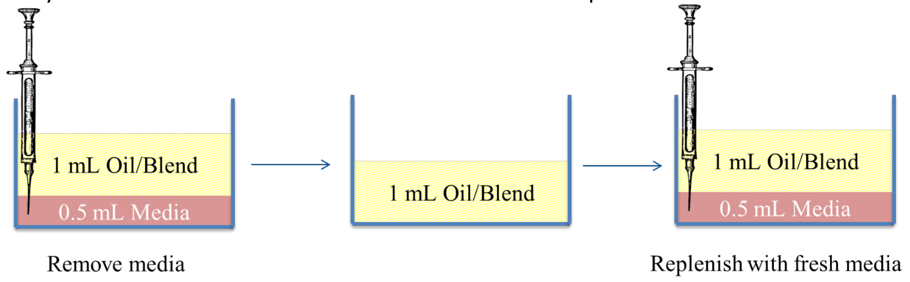

Figure S4 Illustration of experimental setup for release experiments and how media samples were taken throughout drug release studies.

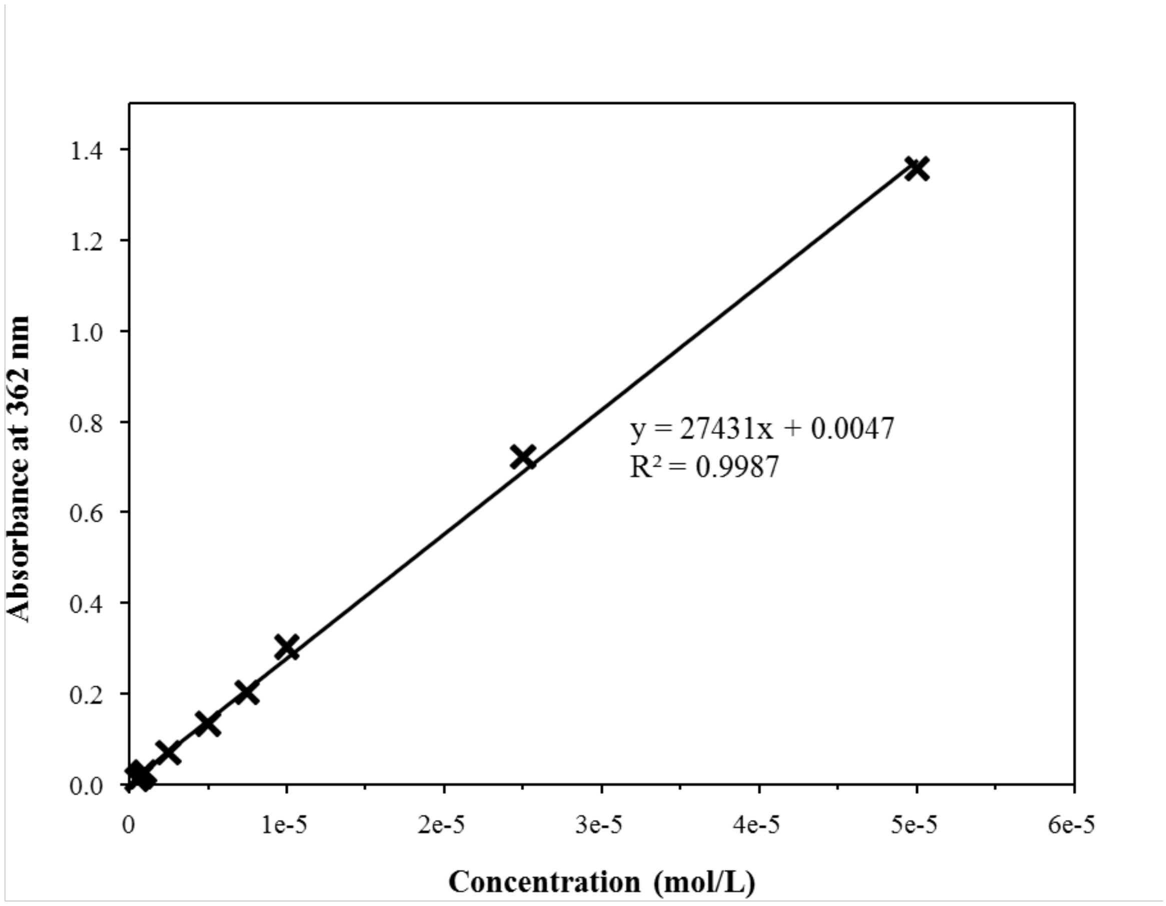

Figure S5 UV-Vis calibration curve of atRA in DMSO:H_2O (8:2) measured at 362 nm wavelength.

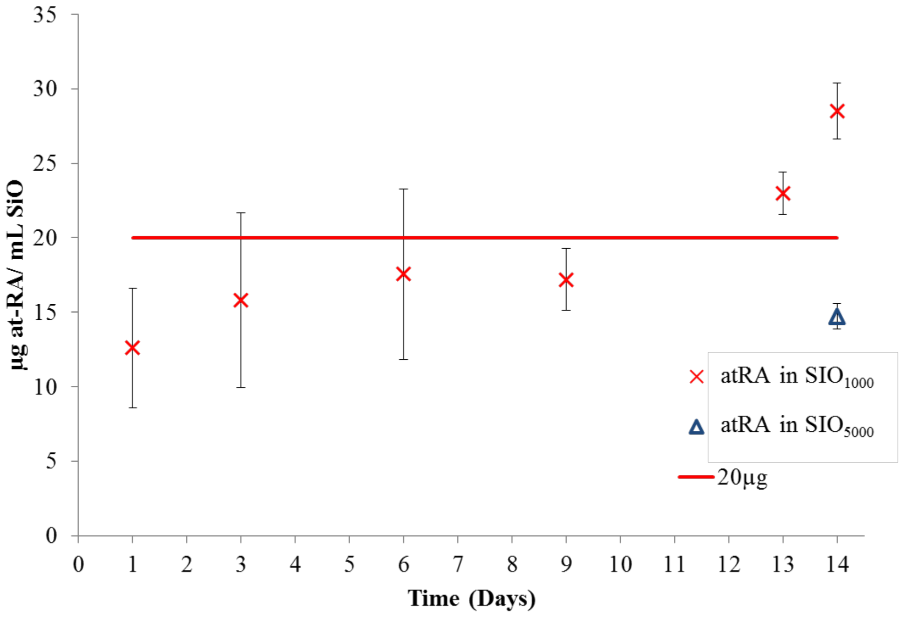

Figure S6 UV-Vis analysis of atRA solubility in SIO_{1000} and SIO_{5000} over 2 weeks (mean ± SD); n=3. 20 µg line shows solubility of atRA reported in the literature.[35]

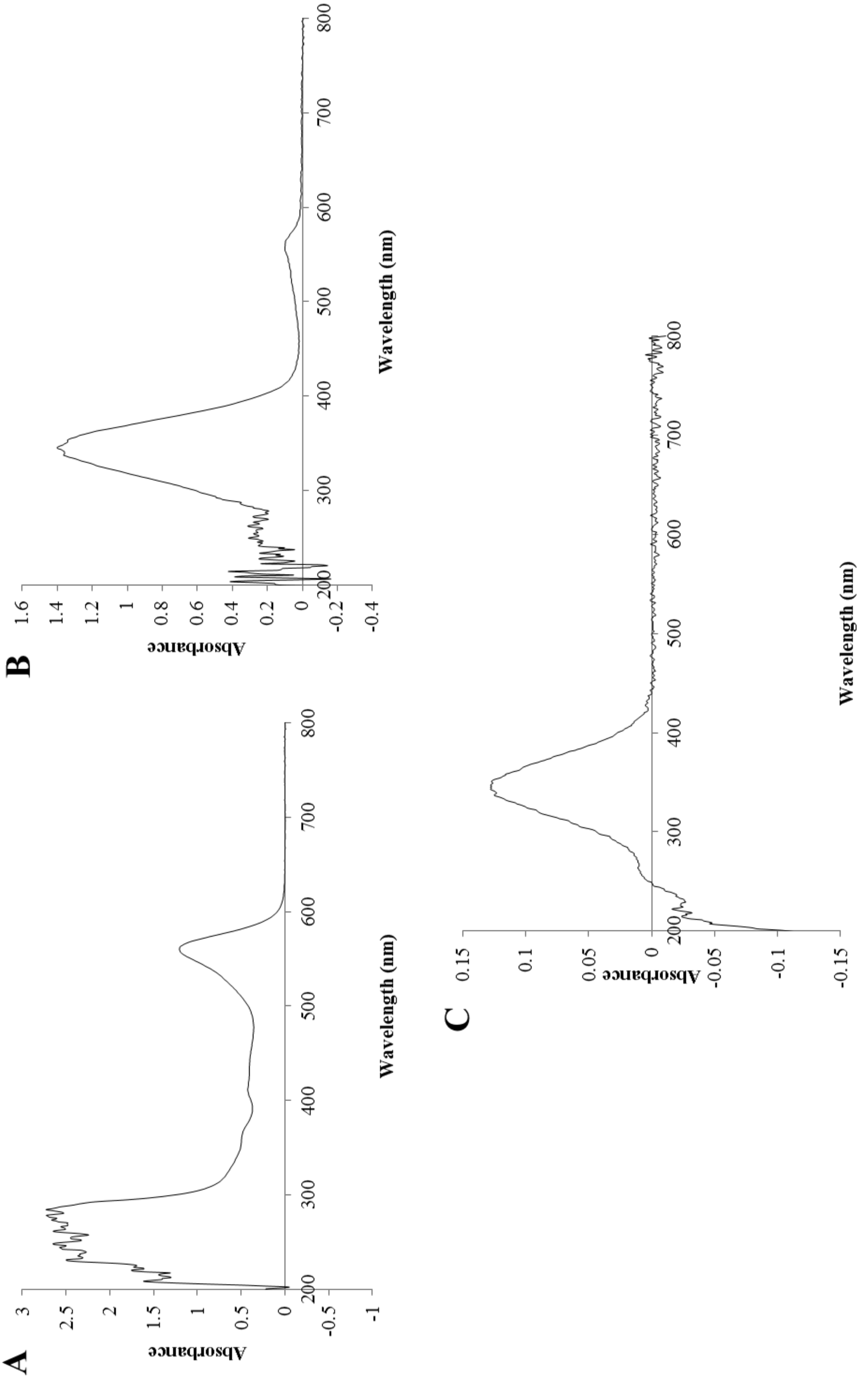

Figure S7 UV-Vis spectra for culture medium with all components (A), atRA $1x10^{-4}$ M in media with all components (B) and atRA $1x10^{-4}$ M in media without FCS (C).

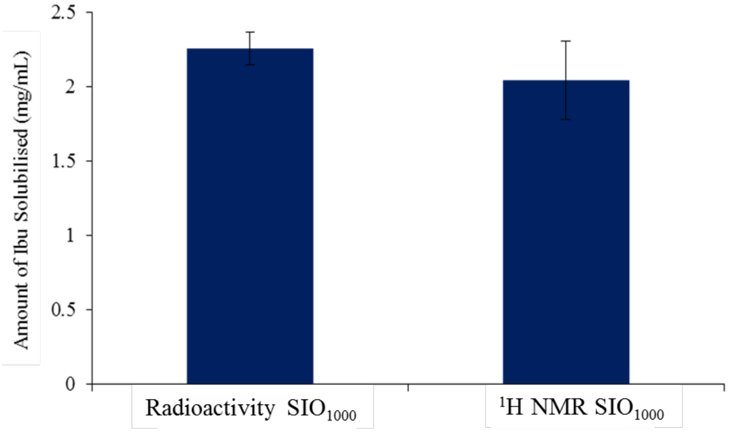

Figure S8 Comparison of saturation concentration of Ibuprofen measured in SIO *via* radiometric analysis (n=4) or ^{1}H NMR ($CDCl_3$, 400 MHz) (n=2) with an internal standard; anisole.

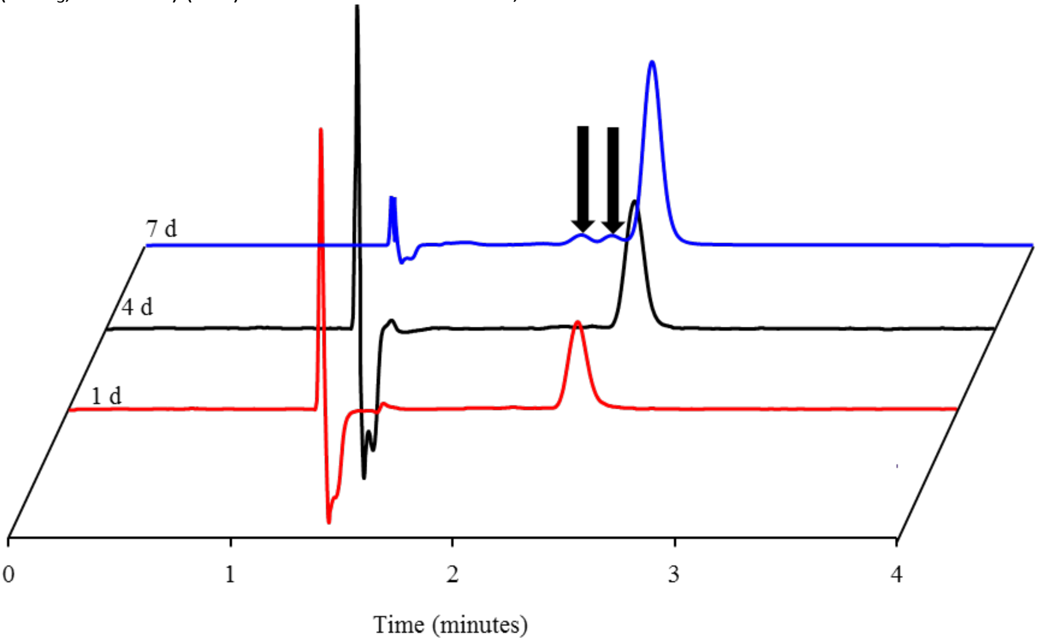

Figure S9 HPLC chromatograms of atRA stored in DCM at ambient temperature in the dark for 1 (red), 4 (black) and 7 (blue) days. (Eluent: 95% ACN; 5% MeOH; 0.6% Acetic Acid). Arrows indicate presence of degradation products

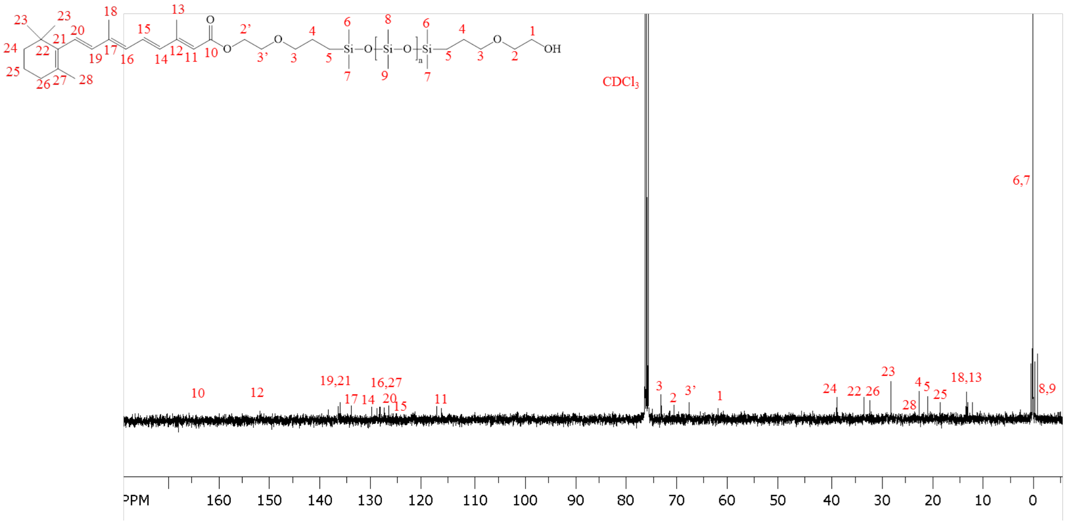

Figure S10 ^{13}C NMR ($CDCl_3$, 100 MHz) atRA-PDMS-atRA.

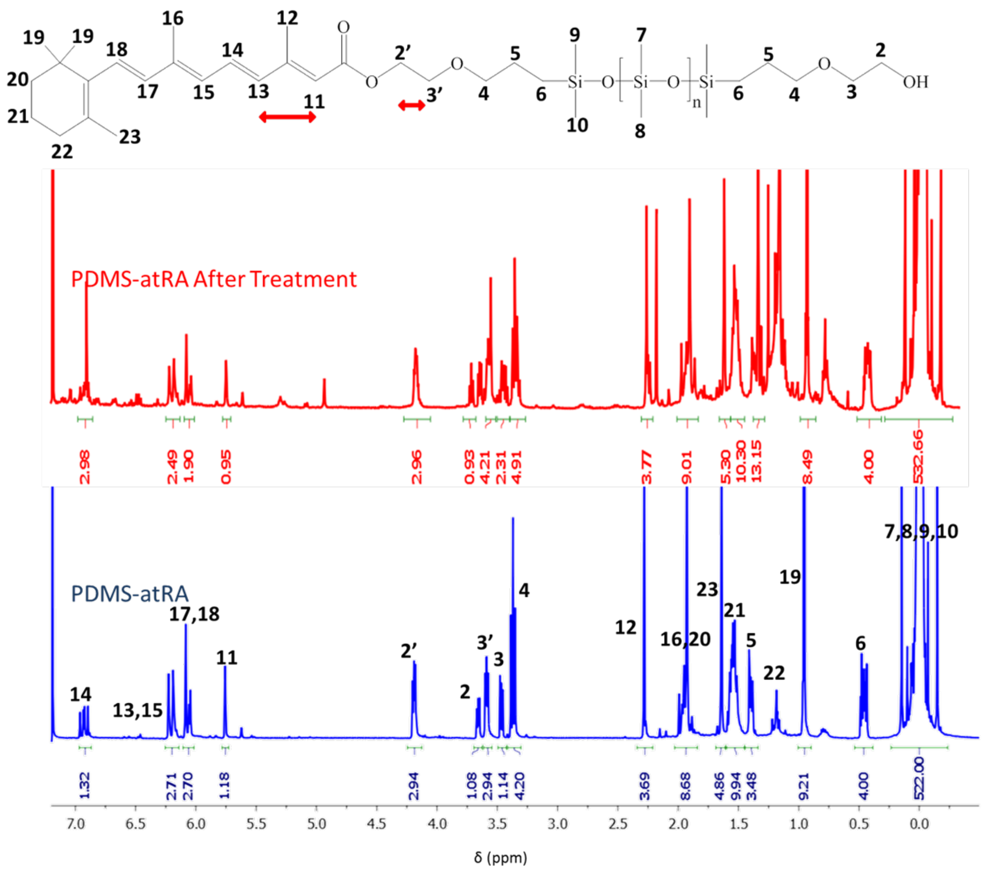

Figure S11 ^{1}H NMR ($CDCl_3$, 400 MHz) PDMS-atRA before (top) and after treatment with 10 equivalents HCl in THF at 40 °C (bottom).

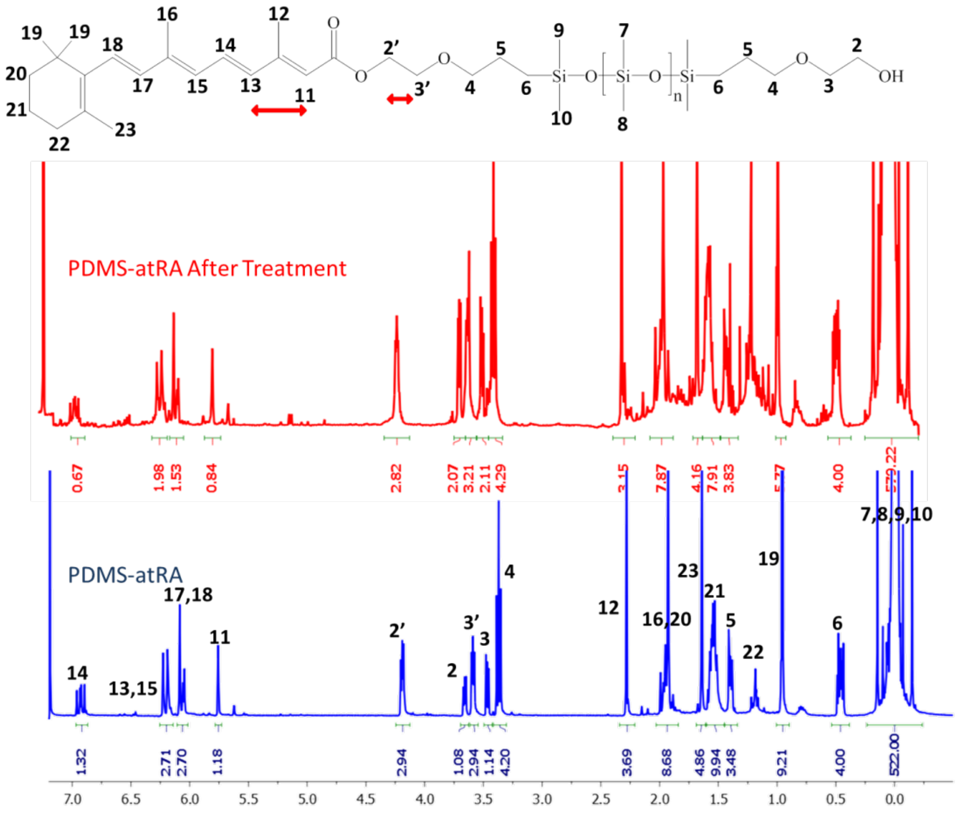

Figure S12 ^{1}H NMR ($CDCl_3$, 400 MHz) PDMS-atRA before (top) and after treatment with 10 equivalents HCl in dioxane at 40 °C (bottom).

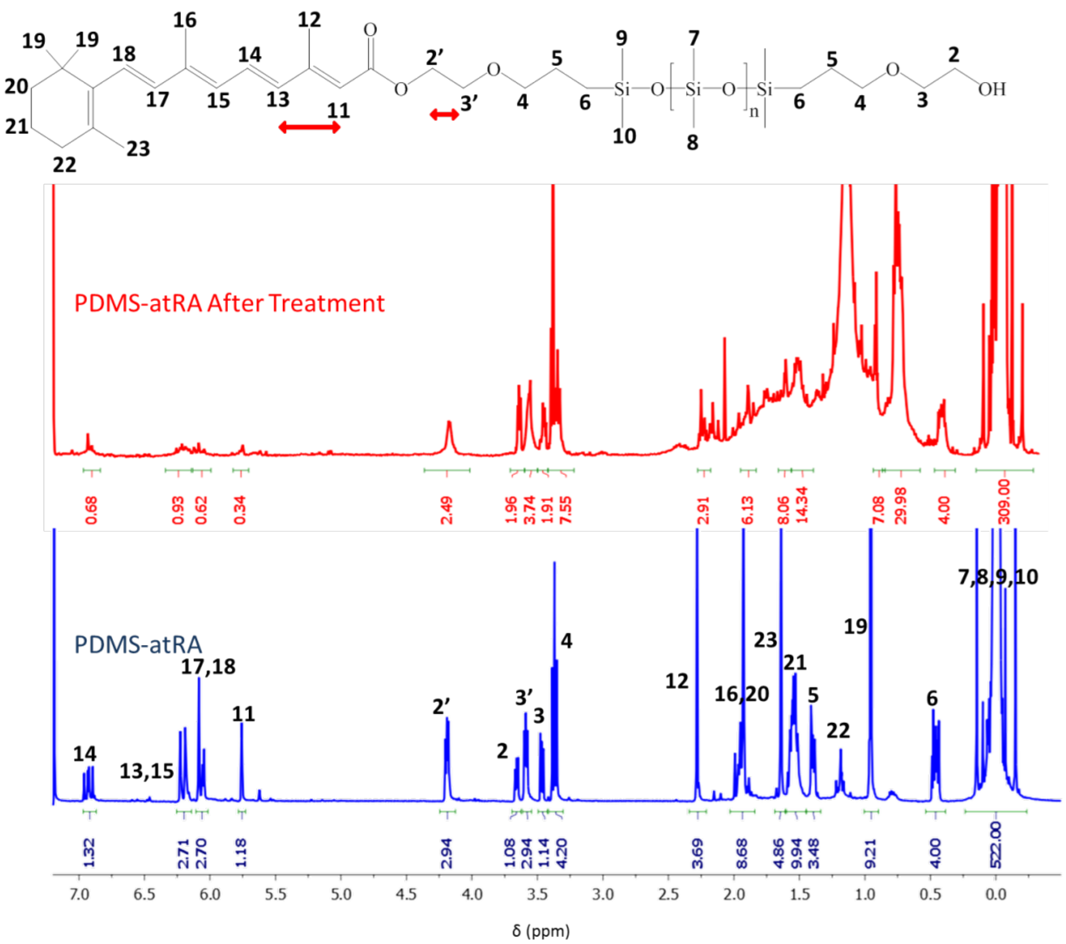

Figure S13 ^{1}H NMR ($CDCl_3$, 400 MHz) PDMS-atRA before (top) and after treatment with 10 equivalents HCl in dioxane at 60 °C (bottom).

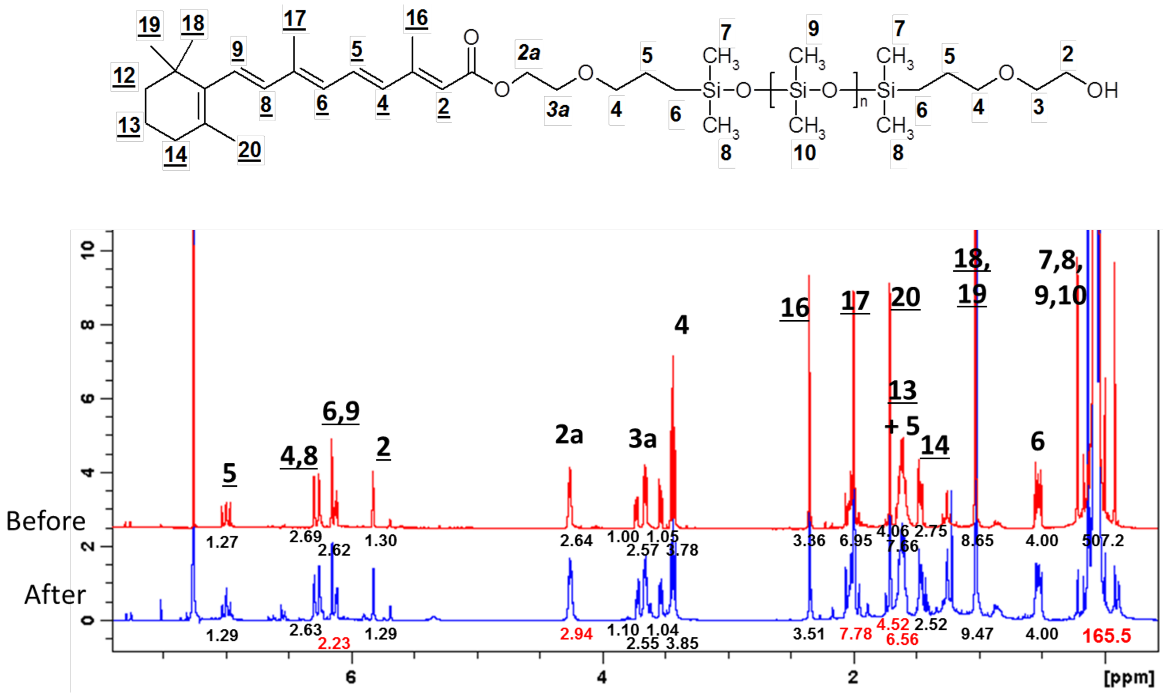

Figure S14 ^{1}H NMR spectra ($CDCl_3$, 400MHz) of PDMS-atRA before and after exposure to KOH in THF at 40 °C for 22 h, displaying no change of the ester peak (2' and 3'), while the integration of the PDMS main chain signal diminished by a factor of 3-4.

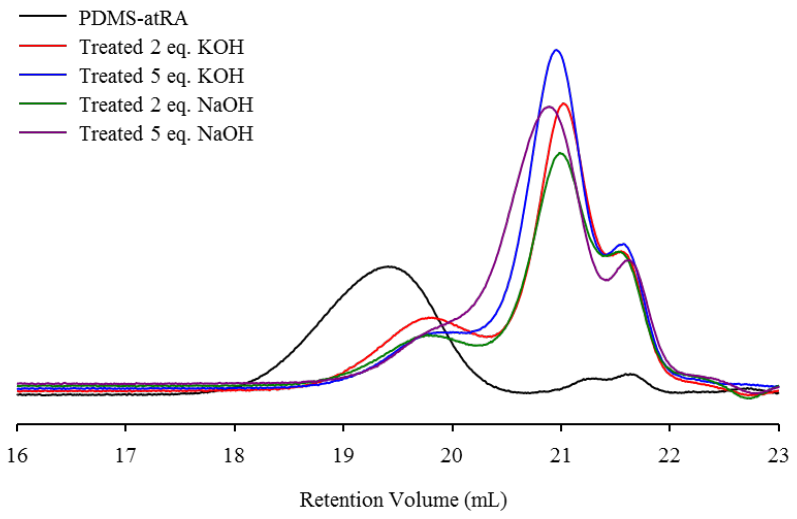

Figure S15 GPC RI chromatogram overlays of PDMS-atRA (black) before and after exposure to KOH (2 and 5 equivalents) and NaOH (2 and 5 equivalents) in THF at 40 °C for 22 h.

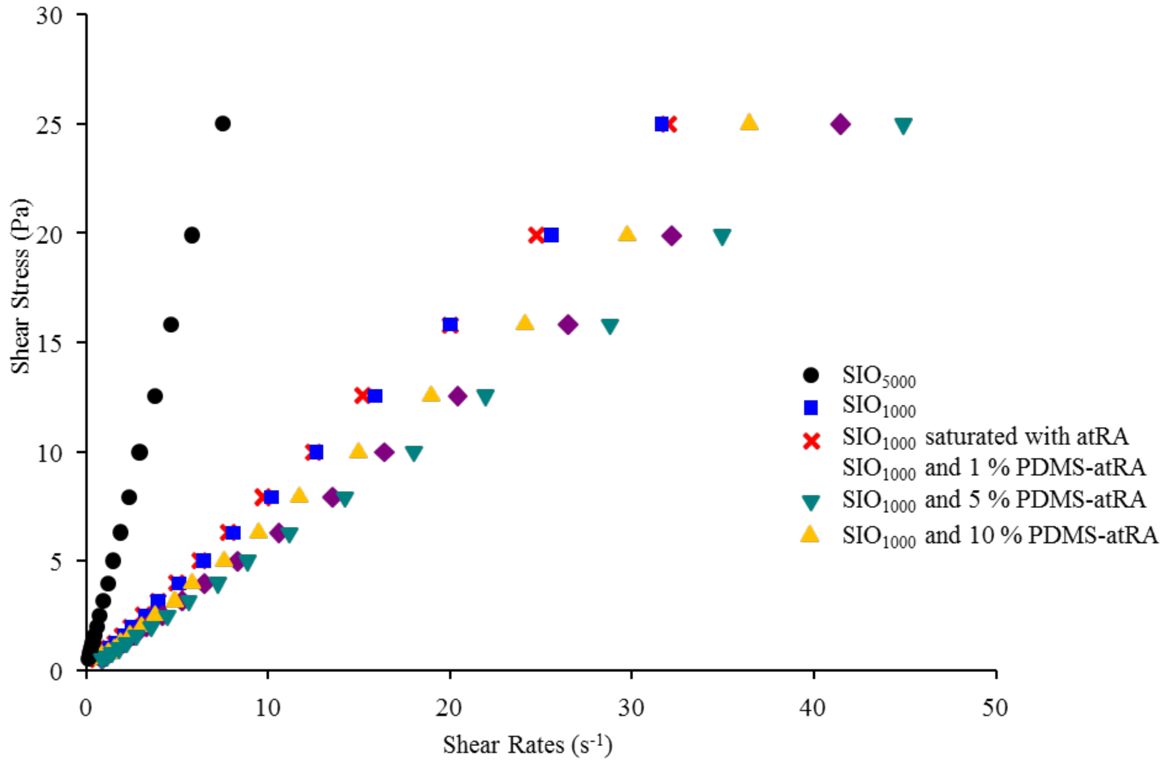

Figure S16 Graph of shear stress against shear rate for SIO_{5000} (●), SIO_{1000} (■), SIO_{1000} saturated with atRA (x), SIO_{1000} blends with 1 (◆), 5(▼) and 10 (▲) % (v/v) PDMS-atRA from which the shear viscosity was calculated.

Table S1 Characteristics of the atRA-PDMS-atRA synthesised and used throughout the project.

Conversion (%)[†]	Average M_n ($gmol^{-1}$)[†]	DP_n[†]	[atRA] 10^{-4} (mol/g)
72	6,810	82	2.11
63	7,060	86	1.78
59	7,040	86	1.67
60	6,960	85	1.72

[†]Determined by ^1H NMR spectroscopy analyses.

Table S2 Shear viscosities (Pa.s) calculated from the gradient of the plot of shear stress against shear rate at 20°C and 37 °C.

Material	Shear Viscosity (Pa.s) at 37 °C	Shear Viscosity (Pa.s) at 20 °C
SIO_{5000}	3.35	4.81
SIO_{1000}	0.79	1.09
SIO_{1000} saturated with atRA	0.79	1.11
SIO_{1000} with 1 % PDMS-atRA	0.61	0.84
SIO_{1000} with 5 % PDMS-atRA	0.68	0.93
SIO_{1000} with 10 % PDMS-atRA	0.56	0.82
